# Genetic Strategies for Labeling AT2 Cells in Murine Lung via Abca3 and Etv5-Driven Reporters

**DOI:** 10.70322/jrbtm.2026.10002

**Published:** 2026-02-06

**Authors:** Xue Liu, Xuexi Zhang, Zekun Li, Vrishika Kulur, Ningshan Liu, Jiurong Liang, Dianhua Jiang

**Affiliations:** 1 Women’s Guild Lung Institute, Department of Medicine, Cedars-Sinai Medical Center, Los Angeles, CA 90048, USA;; 2 Department of Biomedical Sciences, Cedars-Sinai Medical Center, Los Angeles, CA 90048, USA

**Keywords:** Alveolar type 2 (AT2) cells, Abca3, Etv5, Lung development, Bleomycin injury

## Abstract

Precise labeling of alveolar type 2 (AT2) cells is essential for elucidating lung development and injury responses. In this study, we evaluated Abca3 and Etv5-based genetic strategies for labeling AT2 cells in murine models. Using targeted genetic approaches, we generated Abca3-rtTA and Etv5-rtTA knock-in mouse lines and crossed them with pTRE-H2BGFP to create inducible reporter models driven by Abca3 or Etv5. Labeling specificity and efficiency were assessed by flow cytometry and co-immunostaining. Our results show that both Abca3 and Etv5 strategies faithfully label AT2 cells across developmental stages and following lung injury. Comprehensive analyses confirmed the high specificity and efficiency of labeling. These Abca3- and Etv5-driven systems offer robust tools for investigating AT2 cell biology and pathology and may serve as effective drivers for tetO-mediated gene knockout or overexpression studies specifically in AT2 cells in mouse models.

## 1. Introduction

Alveolar type 2 (AT2) epithelial cells play a central role in maintaining lung homeostasis, repair, and regeneration. These cuboidal cells are responsible for synthesizing and secreting pulmonary surfactant, a lipoprotein complex that reduces surface tension and prevents alveolar collapse during respiration [1]. Beyond their classical function in surfactant production, AT2 cells serve as progenitor cells for the alveolar epithelium, capable of self-renewal and differentiation into alveolar type 1 (AT1) cells following injury [2]. Their ability to replenish the alveolar lining after damage positions them as critical mediators of lung repair [3]. Dysregulation of AT2 cell function contributes to a variety of pulmonary diseases, including acute respiratory distress syndrome (ARDS) [4], idiopathic pulmonary fibrosis (IPF) [3], and certain forms of lung cancer [5]. Thus, precise identification and manipulation of AT2 cells are essential for understanding the mechanisms that govern lung development, injury responses, and disease progression.

To investigate AT2 cell biology *in vivo*, lineage tracing and cell-specific gene manipulation strategies rely heavily on the availability of accurate genetic drivers. Traditional AT2 cell labeling approaches have utilized promoters such as Sftpc (surfactant protein C) [6–8] to drive reporter or recombinase expression. Among these, Sftpc-Cre [8] and Sftpc-rtTA [6] mouse lines have been widely used to target AT2 cells due to the selective expression of surfactant protein C. However, these lines are not without limitations. Some Sftpc-based models exhibit ectopic activity in non-AT2 cell types, including distal airway progenitors [7,9], and may show developmental-stage–dependent variability in expression. In addition, continuous or high-level Sftpc-driven recombinase activity can lead to off-target effects or perturbations in AT2 cell physiology [10–12], complicating the interpretation of experimental results. Therefore, identifying alternative promoters that more precisely and consistently label AT2 cells remains a critical need in lung research.

Recent transcriptomic and epigenetic studies have revealed Abca3 (ATP-binding cassette subfamily A member 3) and Etv5 (ETS variant transcription factor 5) as highly specific markers of mature AT2 cells. Abca3 is a key regulator of lamellar body biogenesis and surfactant lipid transport [13,14], with loss-of-function mutations linked to neonatal respiratory failure [15] and interstitial lung disease in children [16]. Etv5, a transcription factor downstream of FGF signaling, is essential for maintaining AT2 cell identity and preventing trans-differentiation into AT1 cells [17]. Both genes are expressed robustly and specifically in AT2 cells throughout development and adulthood, making them promising candidates for generating new genetic labeling tools.

In this study, we developed *Abca3-rtTA* and *Etv5-rtTA* knock-in mouse lines and crossed them with *pTRE-H2BGFP* to create inducible, lineage-specific reporter models. Using flow cytometry and co-immunostaining, we demonstrate that these strategies enable efficient and specific labeling of AT2 cells during various developmental stages and after lung injury. The Abca3- and Etv5-driven systems not only provide reliable models for tracking and isolating AT2 cells but also offer valuable platforms for conditional gene manipulation through tetO-mediated knockout or overexpression approaches. These new genetic tools expand the repertoire of AT2-specific drivers and will facilitate more precise studies of alveolar epithelial cell biology in health and disease.

## 2. Materials and Methods

### 2.1. Generation of Abca3-rtTA and Etv5-rtTA Knock-In Mouse Lines

To develop inducible and AT2-specific reporter models, we generated *Abca3*-rtTA and *Etv5*-rtTA knock-in mouse lines using CRISPR/Cas9-mediated genome editing (Biocytogen, Waltham, MA, USA). The design of targeting constructs was guided by transcriptomic data and by previous studies that identified *Abca3* and *Etv5* as robust AT2-specific markers. For *Abca3*-rtTA knock-in, an internal ribosome entry site (IRES) followed by the reverse tetracycline-controlled transactivator (*rtTA*) cassette was targeted into the junction region of the last coding exon and 3′UTR of mouse Abca3 via sgRNA. This design preserves endogenous gene expression while enabling doxycycline-inducible activation of tetO-controlled transgenes. For *Etv5*-rtTA knock-in, P2A (derived from porcine teschovirus-1 2A) [18] driven the *rtTA* cassette was targeted into the junction region of last coding exon and 3′UTR of mouse *Etv5* via sgRNA, preserving endogenous gene expression while enabling doxycycline-inducible activation of tetO-controlled transgenes. These mouse lines were generated with C57BL/6J mice. To generate inducible reporter mice, *Abca3*-rtTA and *Etv5*-rtTA lines were crossed with *pTRE-H2BGFP* transgenic mice purchased from the Jackson Laboratory (Strain #:0 05104, RRID: IMSR_JAX: 005104), enabling doxycycline-inducible expression of nuclear GFP specifically in AT2 cells. Mice will be placed on a doxycycline diet and water two weeks prior to tissue collection. All animal work was approved by the Institutional Animal Care and Use Committee (IACUC, IACUC008529) at Cedars-Sinai Medical Center and conducted in accordance with NIH guidelines for the care and use of laboratory animals.

### 2.2. Genotyping

Genomic DNA was isolated from tail biopsies using a standard proteinase K digestion method followed by ethanol precipitation or commercially available DNA extraction kits. PCR-based genotyping was performed using primers specific for the targeted *Abca3-rtTA* or *Etv5-rtTA* alleles as well as the *pTRE-H2BGFP* transgene, as listed in Table 1.

### 2.3. Flow Cytometry

Single-cell suspensions from embryonic, postnatal, or adult lungs were prepared as previously described [20]. Lungs were dissected, minced, and digested in a mixture of collagenase type I (2 mg/mL), Dispase (2 mg/mL), and DNase I (100 U/mL) at 37 °C for 30 min with gentle agitation. Digested tissue was filtered through a 70 μm cell strainer, centrifuged at 300× *g* for 5 min, lysed with red blood cell lysis buffer, and resuspended in HBSS containing 2% fetal bovine serum (FBS). Cells were incubated with biotin conjugated primary antibodies (anti-CD31/34/45, eBioscience, San Diego, CA, USA, Cat # 13-0311-85, 13-0341-85, and 13-0451-85, RRIDs: AB_466421, AB_466426, and AB_466447) for 45 min at 4 °C. Fluorescent-conjugated secondary antibody (APC/Cyanine7 Streptavidin, BioLegend, San Diego, CA, USA, Cat # 405208) staining was performed together with surface staining using antibodies against Epcam (PerCP/Cy5.5 anti-CD326, BioLegend, Cat # 118220, RRID: AB_2246499), Sca1 (PE/Cyanine7 anti-Sca1, BioLegend, Cat # 108114, RRID: AB_493596), and CD24 (PE anti-CD24, BioLegend, Cat # 101808, RRID: AB_312841) to identify AT2 cells (Epcam^+^Sca1^−^CD24^−^). Dead cells were excluded using Fixable Viability Dye (eFluor^™^ 780, ThermoFisher Scientific, Waltham, MA, USA, Cat # 65-0865-18). Flow cytometry was performed on a BD LSRFortessa (San Jose, CA, USA). Data were acquired for at least 50,000 live events per sample and analyzed using FlowJo software (v10.8, BD Biosciences, San Jose, CA, USA). GFP+ cells were quantified within the defined AT2 population, and gating strategies were consistently applied across all samples, including littermate controls lacking the rtTA transgenes.

### 2.4. Immunostaining

For immunofluorescence, lungs were inflated with 4% paraformaldehyde (PFA) at 28 cm H_2_O pressure and fixed for 4–24 h at 4 °C. Tissue was then cryoprotected in 30% sucrose overnight, a mixture of 30% sucrose and OCT at 1:1 for 4 h at 4 °C, embedded in OCT, and sectioned at 10 μm thickness. Sections were rinsed in PBS twice in PBS for 29 min, blocked in 5% normal goat serum for 1 h at room temperature, followed by overnight incubation at 4 °C with primary antibodies: anti-Sftpc (Cat # 10774–1-AP, proteintech, Rosemont, IL, USA, RRID: AB_2185497, 1:100) and anti-Pdpn (Cat # 8.1.1, DSHB, Iowa City, IA, USA, RRID: AB_531893, 1:50). After washing, sections were incubated with Alexa Fluor-conjugated secondary antibodies (Cy3 anti-Rabbit antibody, Cat# 111-165-003, Jackson ImmnoResearch Labs, West Grove, PA, USA, RRID: AB_2338000, 1:500 and AF647 anti-Syrian Hamster antibody, Cat # A-21451, AB_2535868, 1:500) for 1.5 h at room temperature. Nuclei were counterstained with DAPI, and slides were mounted with antifade medium. Images were captured using a Zeiss LSM 780 confocal microscope (Zeiss, Jena, Germany) or a similar instrument, with identical acquisition settings across experimental and control samples.

### 2.5. Statistical Analysis

All experiments were conducted with a minimum of three biological replicates unless otherwise indicated. Flow cytometry data were analyzed using FlowJo, and statistical analyses were performed in GraphPad Prism (v9.0). Data are presented as mean ± standard error of the mean (SEM). For multiple comparisons, one-way analysis of variance (ANOVA) followed by Tukey’s post-hoc test was applied as appropriate. Significance levels are indicated in figure legends (* *p* < 0.05, ** *p* < 0.01, *** *p* < 0.001, **** *p* < 0.0001).

## 3. Results

### 3.1. Generation of Abca3- and Etv5-Driven Inducible Reporter Mouse Models

**Figure 1.**
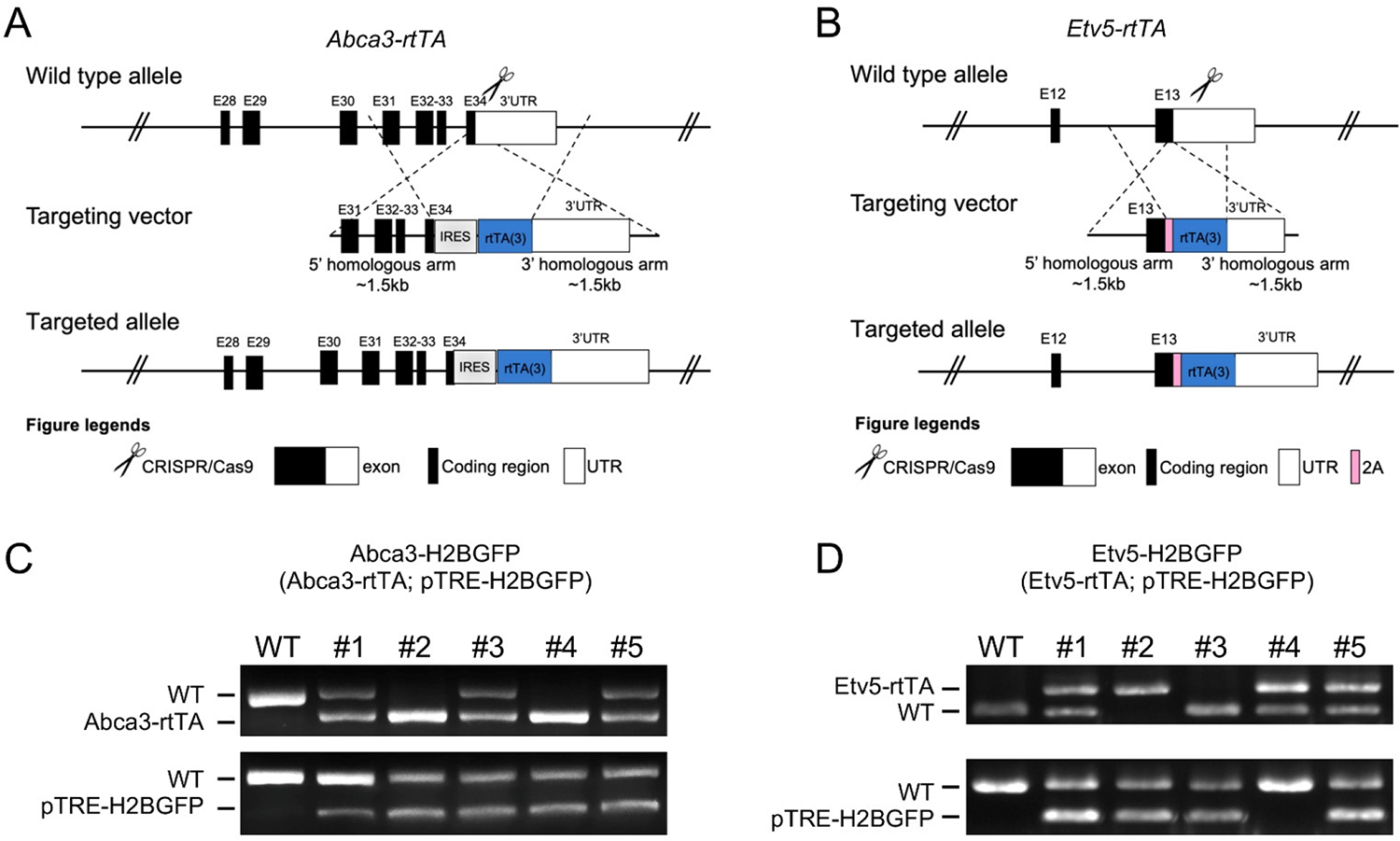
Generation of *Abca3-H2BGFP* and *Etv5-H2BGFP* mouse models. (**A**,**B**) The design strategies of *Abca3-rtTA* (**A**) and *Etv5-rtTA* (**B**) mouse lines. (**C**,**D**) Representative genotyping results of *Abca3-H2BGFP* ((**C**), *Abca3-rtTA; pTRE-H2BGFP*) and *Etv5-H2BGFP* ((**D**), *Etv5-rtTA; pTRE-H2BGFP*) mouse.

Abca3 and Etv5 are commonly used alongside surfactant genes, such as Sftpc, as markers of AT2 cells. Reanalysis of published scRNA-seq data [21] indicates that these genes are highly and specifically expressed in alveolar progenitors during early lung development and in fully differentiated AT2 cells at later stages (Figure S1). To establish precise and inducible tools for *in vivo* labeling of AT2 cells, we generated *Abca3-rtTA* (*C57BL/6J-Abca3*^*tm1(IRES-rtTA)Jid*^*/*) and *Etv5-rtTA* (*C57BL/6J-Etv5*^*tm1(P2A-rtTA)Jid*^*/*) knock-in mouse lines using CRISPR/Cas9-mediated genome editing (Figure 1A,B). In each targeting construct, an IRES-rtTA or P2A-rtTA cassette was integrated into the endogenous locus downstream of the coding sequence and flanked by 5′ and 3′ homology arms to ensure accurate recombination. This bicistronic expression strategy enables doxycycline-inducible transactivation while preserving physiological expression of the native *Abca3* and *Etv5* genes. Both *Abca3-rtTA* and *Etv5-rtTA* mice are viable and exhibit normal lung development without obvious pulmonary abnormalities, indicating that endogenous gene function is largely preserved. Founder animals were bred to *pTRE-H2BGFP* reporter mice [19] to generate the *Abca3-H2BGFP* (*Abca3-rtTA; pTRE-H2BGFP*) and *Etv5-H2BGFP* (*Etv5-rtTA; pTRE-H2BGFP*) dual transgenic lines. PCR genotyping using allele-specific primers confirmed successful rtTA and *TRE-H2BGFP* integration in multiple independent founders, with the absence of the WT band in targeted animals (Figure 1C,D). These data validate the establishment of two inducible, AT2-lineage reporter strains suitable for temporal control of gene expression and fluorescent lineage labeling.

### 3.2. Robust Alveolar Progenitor Cell Labeling in E15.5 Embryonic Lungs

**Figure 2.**
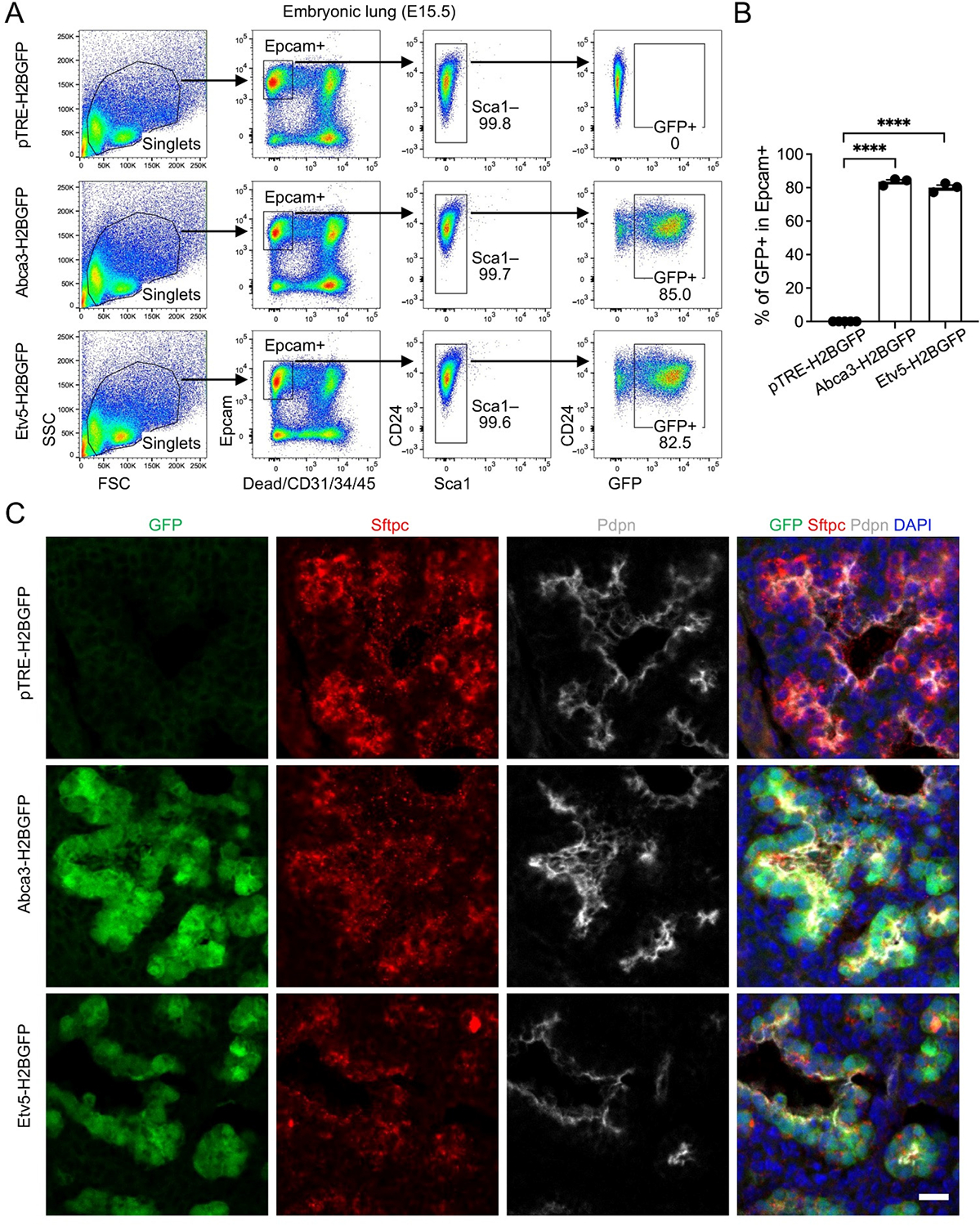
Labeling of alveolar progenitors by the expression of Abca3 and Etv5 in embryonic day E15.5 (E15.5) mouse lungs. (**A**) Representative flow cytometry plots showing the gating strategy for GFP^+^ cells in E15.5 *Abca3-H2BGFP* and *Etv5-H2BGFP* lungs, and littermate controls (*pTRE-H2BGFP*). (**B**) Quantification of the GFP^+^ cells in total Epcam^+^ cells by flow cytometry analysis. *pTRE-H2BGFP*, *n* = 5; *Abca3-H2BGFP*, *n* = 3; *Etv5-H2BGFP*, *n* = 3. **** *p* < 0.0001 by one-way ANOVA. (**C**) Representative co-immunostaining of GFP with AT2 (Sftpc) and AT1 (pdpn) markers in lung sections from E15.5 *Abca3-H2BGFP* and *Etv5-H2BGFP* mice, compared with littermate controls (*pTRE-H2BGFP*). Scale bar: 50 μm.

To assess inducible labeling during early alveolar morphogenesis, doxycycline administration was initiated immediately upon vaginal plug detection and maintained continuously via doxycycline-supplemented chow and drinking water throughout gestation until embryo collection. Embryonic lungs were harvested at E15.5 and analyzed by flow cytometry and immunofluorescence. Within the Epcam^+^ epithelial compartment, GFP expression was observed in over 80% of cells in both *Abca3-H2BGFP* and *Etv5-H2BGFP* lungs, reflecting highly efficient reporter activation, whereas *pTRE-H2BGFP* control littermates exhibited only baseline signal (Figure 2A,B). At this developmental stage, nearly all epithelial cells were CD24^+^, with no detectable Sca1^+^ cells, suggesting that alveolar and airway epithelial populations cannot be reliably distinguished using these markers. The Epcam^+^GFP^−^ fraction likely corresponds to airway epithelial cells. Examination of non-epithelial compartments confirmed the absence of GFP^+^ cells in immune, endothelial, and stromal populations (Figure S2A). A previous study suggested that distal tip progenitor cells persist through ~E17.5 and progressively acquire alveolar (AT1/AT2) markers [22], indicating that Abca3/Etv5-driven labeling at E15.5–E17.5 likely captures a mixed population of tip progenitors and differentiating alveolar cells. Immunostaining of lung sections demonstrated strong co-localization of GFP with the AT2 marker Sftpc, with GFP signal closely adjacent to but largely non-overlapping with the AT1 marker Pdpn, indicating that reporter activation is specifically restricted to tip progenitors and differentiating alveolar cells (Figure 2C). Together, these results demonstrate that both promoter systems are already transcriptionally active in a substantial fraction of developing distal lung epithelium and are capable of selectively marking alveolar progenitor cells (differentiating AT2 cells) at this stage.

### 3.3. Sustained and Lineage-Restricted Labeling at Late Embryonic Stage (E17.5)

**Figure 3.**
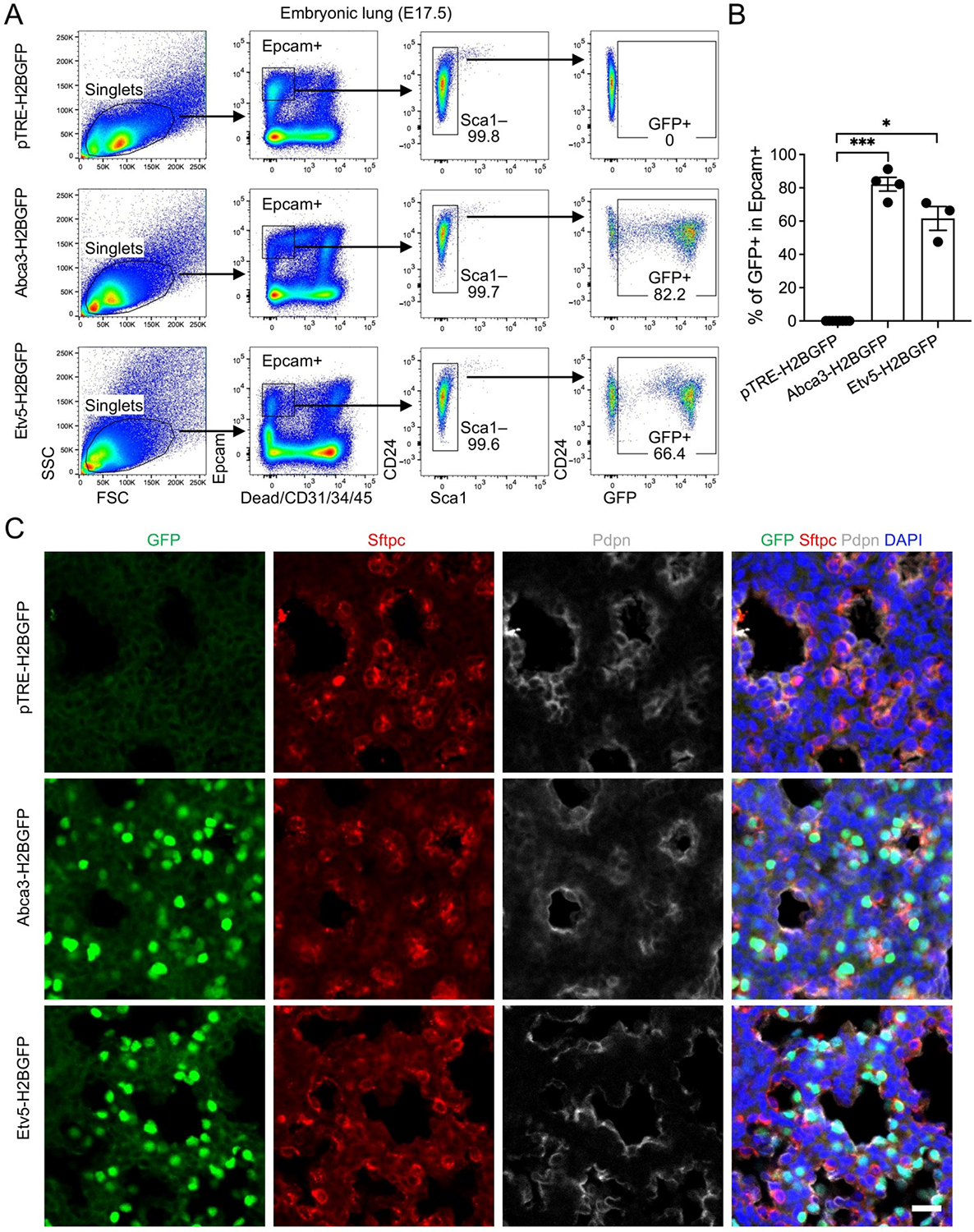
Labeling of alveolar progenitors by the expression of Abca3 and Etv5 in embryonic day E17.5 (E17.5) mouse lungs. (**A**) Representative flow cytometry plots showing the gating strategy for GFP^+^ cells in E17.5 *Abca3-H2BGFP* and *Etv5-H2BGFP* lungs, and littermate controls (*pTRE-H2BGFP*). (**B**) Quantification of the GFP^+^ cells in total Epcam^+^ cells by flow cytometry analysis. *pTRE-H2BGFP*, *n* = 8; *Abca3-H2BGFP*, *n* = 4; *Etv5-H2BGFP*, *n* = 3. * *p* < 0.05, *** *p* < 0.001 by one-way ANOVA. (**C**) Representative co-immunostaining of GFP with AT2 (Sftpc) and AT1 (pdpn) markers in lung sections from E17.5 *Abca3-H2BGFP* and *Etv5-H2BGFP* mice, compared with littermate controls (*pTRE-H2BGFP*). Scale bar: 50 μm.

To determine whether reporter activity is maintained during late gestation, we analyzed E17.5 lungs, a stage characterized by active surfactant production and ongoing AT2 lineage specification. Flow cytometry revealed GFP expression in over 80% of Epcam^+^ cells in *Abca3-H2BGFP* lungs and approximately 70% in *Etv5-H2BGFP* lungs, both markedly higher than the background signal in control mice (Figure 3A,B). As observed at E15.5, all Epcam^+^ epithelial cells were CD24^+^, with no detectable Sca1^+^ populations, indicating that alveolar and airway epithelial cells remain indistinguishable by these markers at this stage. Immunostaining confirmed strong co-localization of GFP with Sftpc^+^ alveolar progenitor epithelial cells, with minimal overlap with the AT1 marker Pdpn, demonstrating that reporter induction remains highly selective for the differentiating AT2 cells (Figure 3C). The slightly lower labeling efficiency in Etv5-H2BGFP lungs likely reflects developmental stage–dependent differences in promoter activity, potentially corresponding to temporal fluctuations in endogenous Etv5 expression during late alveologenesis.

### 3.4. High-Efficiency AT2 Labeling in Early Postnatal Lungs (P7)

**Figure 4.**
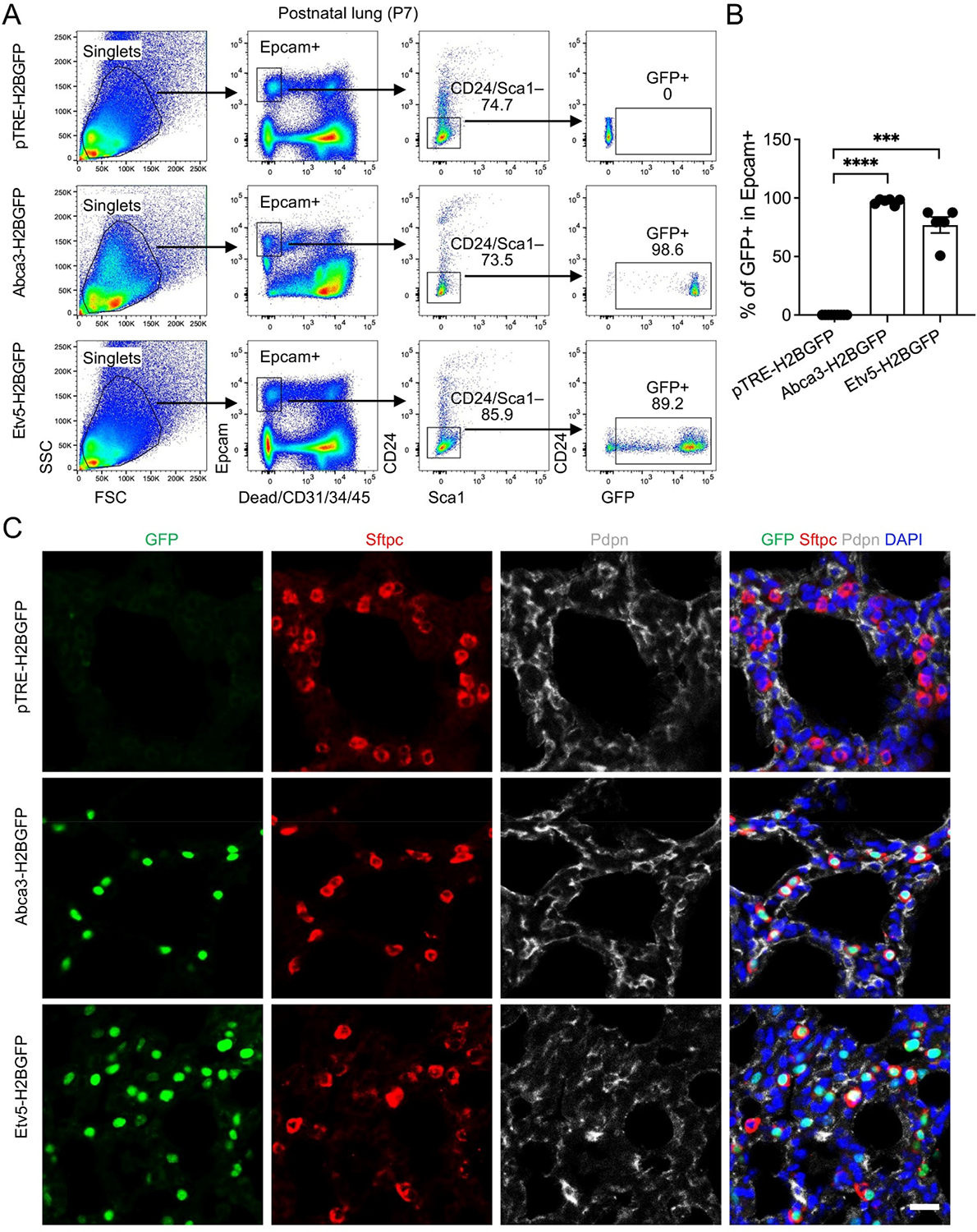
Labeling of AT2 cells by the expression of Abca3 and Etv5 on Postnatal day 7 (P7) mouse lungs. (**A**) Representative flow cytometry plots showing the gating strategy for GFP^+^ cells in P7 *Abca3-H2BGFP* and *Etv5-H2BGFP* lungs, and littermate controls (*pTRE-H2BGFP*). (**B**) Quantification of the GFP^+^ cells in total Epcam^+^Sca-1^−^CD24^−^ AT2 cells by flow cytometry analysis. *pTRE-H2BGFP*, *n* = 9; *Abca3-H2BGFP*, *n* = 6; *Etv5-H2BGFP*, *n* = 5. *** *p* < 0.001, **** *p* < 0.0001 by one-way ANOVA. (**C**) Representative co-immunostaining of GFP with AT2 (Sftpc) and AT1 (pdpn) markers in lung sections from P7 *Abca3-H2BGFP* and *Etv5-H2BGFP* mice, compared with littermate controls (*pTRE-H2BGFP*). Scale bar: 50 μm.

To evaluate reporter performance during early postnatal alveolar expansion, we analyzed lungs at postnatal day 7 (P7). AT2 cells were identified using a refined flow cytometry gating strategy (Epcam^+^Sca1^−^CD24^−^), which reliably isolates the alveolar epithelial population at this developmental stage. GFP expression was observed in 98.6% of AT2 cells in *Abca3-H2BGFP* mice and 89.2% in *Etv5-H2BGFP* mice, reflecting near-complete labeling of the AT2 niche and demonstrating the high efficiency of both inducible reporter systems (Figure 4A,B). Analysis of non-epithelial fractions showed an absence of GFP^+^ cells in immune, endothelial, and stromal compartments, with only rare GFP^+^ events (Figure S2B)–likely attributable to incomplete Epcam staining or cell contaminants/doublets during flow cytometry recording. Histological analyses further validated these findings, showing robust nuclear GFP signal localized specifically to Sftpc^+^ AT2 cells, with minimal to no expression in neighboring Pdpn^+^ AT1 cells or other epithelial subsets (Figure 4C). These results indicate that both Abca3- and Etv5-driven reporters achieve highly penetrant, lineage-restricted labeling, capturing the vast majority of the AT2 compartment. Collectively, this highlights their utility for fate-mapping and functional studies during a critical window of postnatal alveolar development, when AT2 cells are actively expanding and maturing.

### 3.5. Efficient AT2 Targeting in Adult Lungs under Homeostasis and Early after Injury

**Figure 5.**
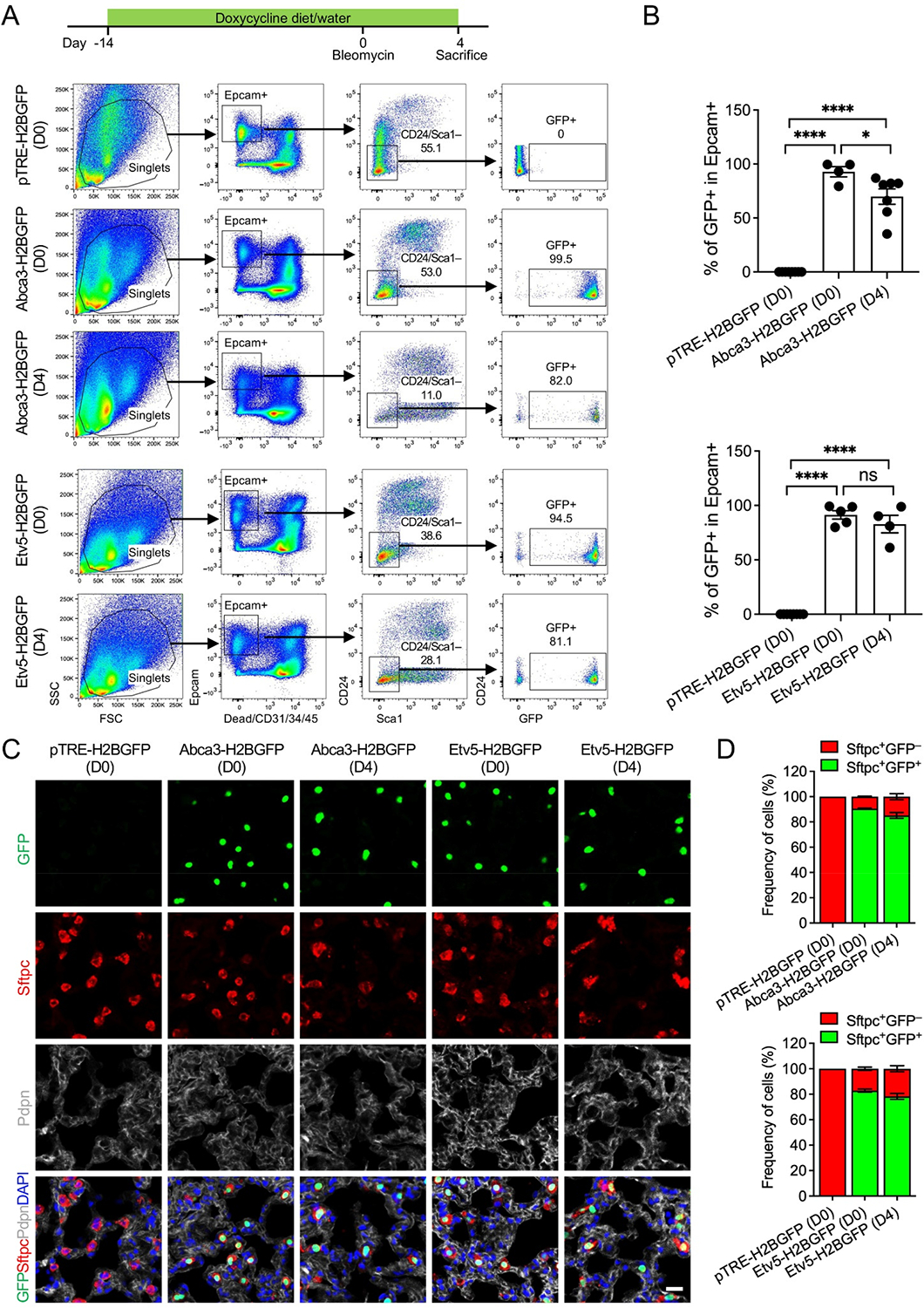
Labeling of AT2 cells by the expression of Abca3 and Etv5 in adult mouse lungs before and after bleomycin injury. (**A**) Representative flow cytometry plots showing the gating strategy for GFP^+^ cells in lungs from adult *Abca3-H2BGFP* and *Etv5-H2BGFP*, and littermate control (*pTRE-H2BGFP*) mice before (D0) and 4 days after bleomycin injury (D4). (**B**) Quantification of the GFP^+^ cells in total Epcam^+^ Sca-1^−^CD24^−^ AT2 cells by flow cytometry analysis. *pTRE-H2BGFP* (D0), *n* = 8; *Abca3-H2BGFP* (D0), *n* = 4; *Abca3-H2BGFP* (D4), *n* = 7, *Etv5-H2BGFP* (D0), *n* = 5; *Etv5-H2BGFP* (D4), *n* = 4. ns, not significant, * *p* < 0.05, **** *p* < 0.0001 by one-way ANOVA. (**C**) Representative co-immunostaining of GFP with AT2 (Sftpc) and AT1 (pdpn) markers in lung sections from adult *Abca3-H2BGFP* and *Etv5-H2BGFP*, and littermate control (*pTRE-H2BGFP*) mice before (D0) and 4 days after bleomycin injury (D4). Scale bar: 50 μm. (**D**) Quantification of the ratios of Sftpc^+^GFP^+^ and Sftpc^+^GFP^−^ cells in lung sections from adult *Abca3-H2BGFP* and *Etv5-H2BGFP*, and littermate control (*pTRE-H2BGFP*) mice before (D0) and 4 days after bleomycin injury (D4).

To evaluate reporter stability in adult lungs and during acute epithelial injury, we profiled mice at baseline (D0) and day 4 following intratracheal bleomycin administration (D4), a time point representing peak inflammatory signaling and early epithelial damage. Under homeostatic conditions, GFP was detected in 99.5% of AT2 cells in *Abca3-H2BGFP* mice and 94.5% in *Etv5-H2BGFP* mice, confirming sustained, near-saturating labeling efficiency in the mature AT2 compartment (Figure 5A,B). Notably, these labeling efficiencies closely mirror those obtained using the tamoxifen-inducible *Sftpc-CreER; Rosa26-tdTomato* system [3] (Figure S3), highlighting the comparability of these doxycycline-responsive models and supporting their utility as fully accessible alternatives for adult AT2 cell tracking and lineage analysis. Direct comparison with the *Sftpc-rtTA* transgenic line [6] was not pursued, as human SFTPC promoter–driven reporters have been reported to exhibit off-target expression in airway epithelial cells [23], limiting their specificity for AT2-restricted labeling. Following bleomycin injury, GFP labeling remained highly preserved, marking 82.0% (Abca3) and 81.1% (Etv5) of AT2 cells despite early injury-induced cellular stress and partial epithelial attrition (Figure 5A,B). Again, examination of non-epithelial compartments showed no substantive GFP signal in immune, endothelial, or stromal cells, with only rare GFP^+^ events detected (Figure S4), most likely reflecting technical artifacts such as incomplete Epcam staining or cell contaminants/doublets during flow cytometric processing. Immunostaining confirmed that GFP remained tightly co-localized with Sftpc^+^ cells both at baseline and post-injury (Figure 5C,D), demonstrating that promoter specificity is maintained even during acute epithelial remodeling. The observed reduction in GFP^+^ frequency after injury likely reflects known early responses, including AT2 apoptosis, transient dedifferentiation, and plasticity of the epithelial cell state, rather than a loss of promoter fidelity.

### 3.6. Advantages and Limitations

Together, these findings demonstrate that *Abca3*- and *Etv5*-driven H2BGFP reporters efficiently and specifically label AT2 cells in adult lungs, maintaining high labeling efficiency and specificity under both homeostatic and early post-injury conditions. These models thus provide versatile and reliable tools for studying AT2 cell biology *in vivo*, offering potential applications for investigating gene function, cellular dynamics, and regenerative mechanisms in the context of adult lung injury and disease. A major strength of these systems is their robust, doxycycline-inducible labeling across developmental stages, from embryonic lung morphogenesis through postnatal alveolar expansion and adult homeostasis. The high penetrance and lineage restriction of GFP expression enable precise tracking and isolation of AT2 cells with minimal off-target labeling in non-epithelial compartments. Moreover, the preservation of endogenous Abca3 and Etv5 function in the knock-in designs supports physiological relevance without overt developmental defects.

However, several limitations should be considered. During early embryogenesis, Abca3- and Etv5-driven labeling captures a mixed population of distal tip progenitors and differentiating alveolar cells, limiting precise discrimination of early lineage states. Labeling efficiency is also modestly reduced following acute lung injury, likely reflecting AT2 cell loss or transient phenotypic changes. In addition, doxycycline-dependent systems require sustained exposure, which may introduce variability in induction timing and levels. In addition, although both Abca3-rtTA and Etv5-rtTA mice were viable and exhibited normal lung development without obvious pulmonary abnormalities under our experimental conditions, prior studies have reported potential rtTA-associated toxicity in certain transgenic contexts [24], which should be considered when interpreting results from Tet-On–based systems. Despite these considerations, the overall specificity, efficiency, and temporal control of these reporters make them powerful tools for AT2-focused studies across lung development, homeostasis, and injury.

## 4. Discussion

AT2 cells are central to maintaining alveolar homeostasis, mediating repair after injury, and serving as progenitors for AT1 cells [2]. Precise genetic labeling of AT2 cells has been critical for dissecting their lineage dynamics during lung development, postnatal maturation, and in response to injury or disease [3]. Traditional labeling approaches, such as Sftpc-Cre or Sftpc-rtTA lines, have enabled lineage tracing and conditional gene manipulation but are limited by developmental-stage-specific expression and occasional off-target activity in distal airway progenitors. Our study introduces *Abca3-rtTA* and *Etv5-rtTA* knock-in mouse lines as complementary tools for AT2 cell labeling. Both genes are highly enriched in AT2 cells throughout development and adulthood, and the inducible nature of the rtTA system allows temporal control over reporter expression. Using these models, we demonstrated robust and specific labeling of AT2 cells during embryonic stages (E15.5, E17.5), early postnatal development (P7), and in adult lungs, including under injury conditions induced by bleomycin. The high labeling efficiency and specificity of these systems make them valuable tools for studying AT2 biology in both homeostatic and pathological contexts, enabling more refined lineage-tracing studies and mechanistic investigations into alveolar epithelial dynamics.

Beyond lineage tracing, the *Abca3*- and *Etv5*-driven rtTA systems provide versatile platforms for conditional gain or loss-of-function studies in AT2 cells through tetO-mediated transgene regulation. By crossing these rtTA lines with tetO-driven constructs, researchers can selectively manipulate gene expression in AT2 cells with temporal control using doxycycline administration. For example, in our recent work [25], we found that ZIP8 is critical for AT2 progenitor function, regulating self-renewal and differentiation, and that its deficiency impairs lung epithelial regeneration, promoting fibrosis [26]. To confirm this finding, we may consider generating a tetO-Zip8 system crossed with an *Abca3*-rtTA or *Etv5*-rtTA line to investigate the role of Zip8 in AT2 cell function during lung injury. Conditional overexpression of Zip8 in AT2 cells allowed us to dissect its impact on epithelial repair, inflammation, and fibrosis in a controlled temporal window.

Fibroblast growth factor (FGF) signaling plays a central role in lung morphogenesis, epithelial cell maintenance, and injury-induced repair, with FGFR2 representing a key receptor mediating AT2 cell proliferation, survival, and regenerative competence [27,28]. Leveraging the inducible and AT2-restricted expression of Abca3-rtTA or Etv5-rtTA, these lines can be crossed with a tetO-FGFR2-HFc dominant-negative mouse strain [29] (JAX #025672) to achieve temporally controlled inhibition of FGF signaling specifically in AT2 cells *in vivo*. This approach enables functional dissection of FGF-dependent processes in discrete biological contexts, including postnatal alveolar maturation, adult tissue homeostasis, and epithelial repair following lung injury. Inducible FGFR blockade in AT2 cells would allow investigators to determine how FGF signaling influences progenitor self-renewal, lineage plasticity, and crosstalk with niche populations such as fibroblasts, endothelial cells, and immune effectors.

Another potential application of these lines is in lung cancer research. For example, the *Abca3-* or *Etv5-rtTA* mice could be crossed with a tetO-Kras^G12D^ responder mouse line [30] (JAX #004375) to generate a model of lung adenocarcinoma in which Kras is selectively and continuously activated in AT2 cells under doxycycline control. This approach allows researchers to precisely regulate oncogene expression temporally and spatially, providing a controlled system to study the initiation, progression, and cellular dynamics of lung tumors. By enabling AT2-specific oncogene activation, these models can also be used to investigate interactions between transformed epithelial cells and the lung microenvironment, test targeted therapies, and dissect mechanisms of tumorigenesis at specific developmental or adult stages. This demonstrates the broad utility of Abca3- and Etv5-rtTA lines beyond homeostasis and injury models, extending their relevance to lung cancer modeling and translational studies.

Despite the robust labeling observed, these models have inherent limitations. Notably, labeling efficiency is not 100%, as a small fraction of AT2 cells do not express *Abca3* or *Etv5* at detectable levels. This heterogeneity may reflect developmental-stage-specific expression, regional differences within the alveolar epithelium, or transient transcriptional states of AT2 cells. Consequently, a subset of AT2 cells may escape labeling, potentially limiting the interpretation of lineage-tracing experiments or conditional gene manipulation in studies requiring complete targeting of the AT2 population. Additionally, while doxycycline-inducible systems allow temporal control, incomplete induction or variability in doxycycline delivery could further contribute to heterogeneity in reporter expression. Researchers should consider these factors when designing experiments, particularly in studies where precise quantification of the entire AT2 population is critical. Future refinements, such as combining multiple AT2-specific promoters or optimizing induction protocols, may help mitigate these limitations and further enhance the utility of these models.

## 5. Conclusions

In summary, the *Abca3*- and *Etv5*-rtTA knock-in mouse lines provide highly specific, inducible, and versatile tools for labeling and manipulating AT2 cells across development, postnatal maturation, and adult lung injury. When combined with tetO-mediated transgene systems, these lines enable targeted gain or loss-of-function studies in AT2 cells, facilitating mechanistic investigations into lung repair and disease. While not every AT2 cell is labeled, the high efficiency and fidelity of these models make them powerful platforms for advancing our understanding of alveolar epithelial dynamics and for informing therapeutic strategies targeting AT2-mediated repair.

## Supplementary Material

Supplementary Inforation

The following supporting information can be found at: https://www.sciepublish.com/article/pii/870, Figure S1: Transcriptional of Sftpc, Abca3, and Etv5 in embryonic and postnatal mouse lungs by published single cell RNA-seq dataset GSE149563; Figure S2: Representative flow cytometry plots showing the gating strategy for GFP^+^ cells in CD31/34/45^+^ mixed immune/endothelial cells and in double negative stromal cells in embryonic (**A**, E15.5) and postnatal (**B**, P7) *Abca3-H2BGFP* and *Etv5-H2BGFP* lungs, and littermate controls (*pTRE-H2BGFP*); Figure S3: Representative flow cytometry plots showing the gating strategy for tdTomato^+^ cells in lungs from uninjured adult *Sftpc-CreER*; *Rosa26-tdTomato* and littermate control (*Rosa26-tdTomato*) mice after Tamoxifen induction; Figure S4: Representative flow cytometry plots showing the gating strategy for GFP^+^ cells in CD31/34/45^+^ mixed immune/endothelial cells and in double negative stromal cells in lungs from adult *Abca3-H2BGFP* and *Etv5-H2BGFP*, and littermate control (*pTRE-H2BGFP*) mice before (D0) and 4 days after bleomycin injury (D4).

## Figures and Tables

**Table 1.** Primer sequences and predicted product sizes.

Transgene	Primer Sequences (5′–3′)	Product Size (bp)
*Abca3-rtTA*	Forward: GGATGATTACTCTGTGAGCCAGATC	WT: 434 Mut: 296
	Reverse (WT): CTGGGGTCTCTCTGGATAAGCACTG	
	Reverse (Mut): AGACCTTGCATTCCTTTGGCGAGAG	
*Etv5-rtTA*	Forward: GTGTGATCTGTCTTCTCACGCAGGT	WT: 314 Mut: 511
	Reverse (WT): ACCACTGCCCTCGGTTGCCTGGATG	
	Reverse (Mut): CAGGAGTGGGTATGATGCCTGTCCA	
*pTRE-H2BGFP* [19]	Forward (WT): AGTGGCCTCTTCCAGAAATG	WT: 521 Mut: 250
	Reverse (WT): TGCGACTGTGTCTGATTTCC	
	Forward (Mut): GCTCGTTTAGTGAACCGTCAG	
	Reverse (Mut): TCTTCTGCGCCTTAGTCACC	

## Data Availability

Mouse lines generated in this study will be deposited to the Mutant Mouse Resource & Research Centers (MMRRC) and will be made available on request by contacting the corresponding author (dianhua.jiang@cshs.org).
