# Supplementary material for: Genetic Strategies for Labeling AT2 Cells in Murine Lung via Abca3 and Etv5-Driven Reporters
Source: J Respir Biol Transl Med. Author manuscript; Available in PMC 2026 Mar 28. (PMC13025979; doi:10.70322/jrbtm.2026.10002)
Supplement: Supplementary Inforation [file NIHMS2155087-supplement-Supplementary_Inforation.pdf]

Figure S1

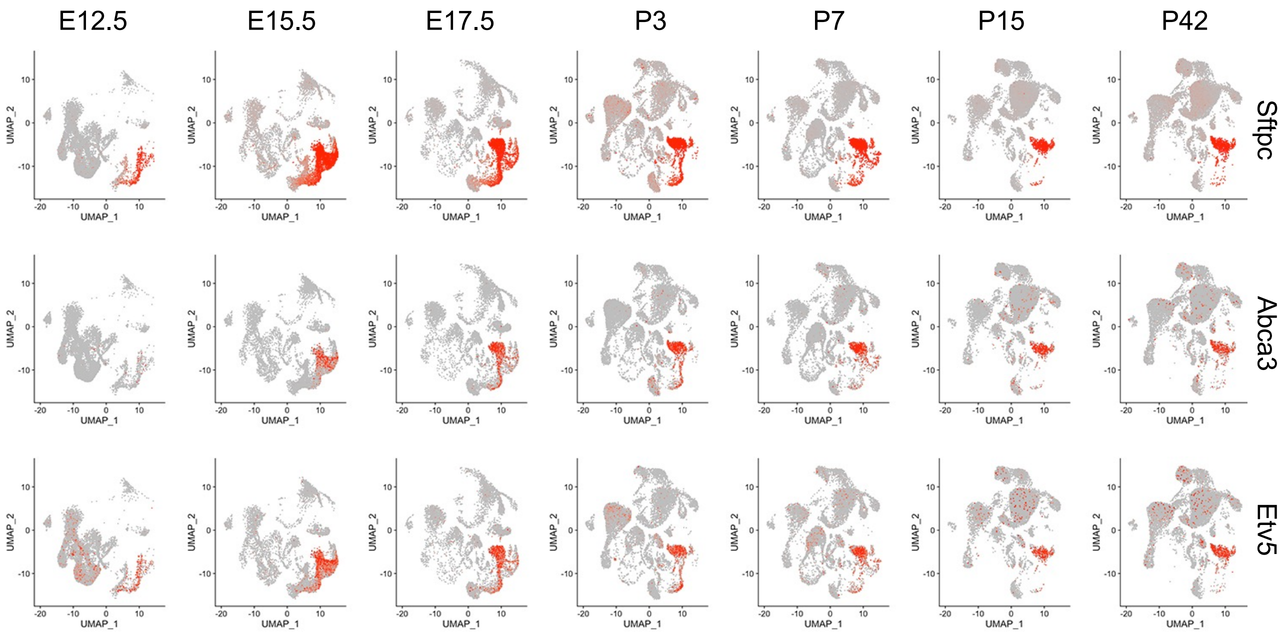

Figure S1. Transcriptional of Sftpc, Abca3, and Etv5 in embryonic and postnatal mouse lungs by published single cell RNA-seq dataset GSE149563.

Figure S2

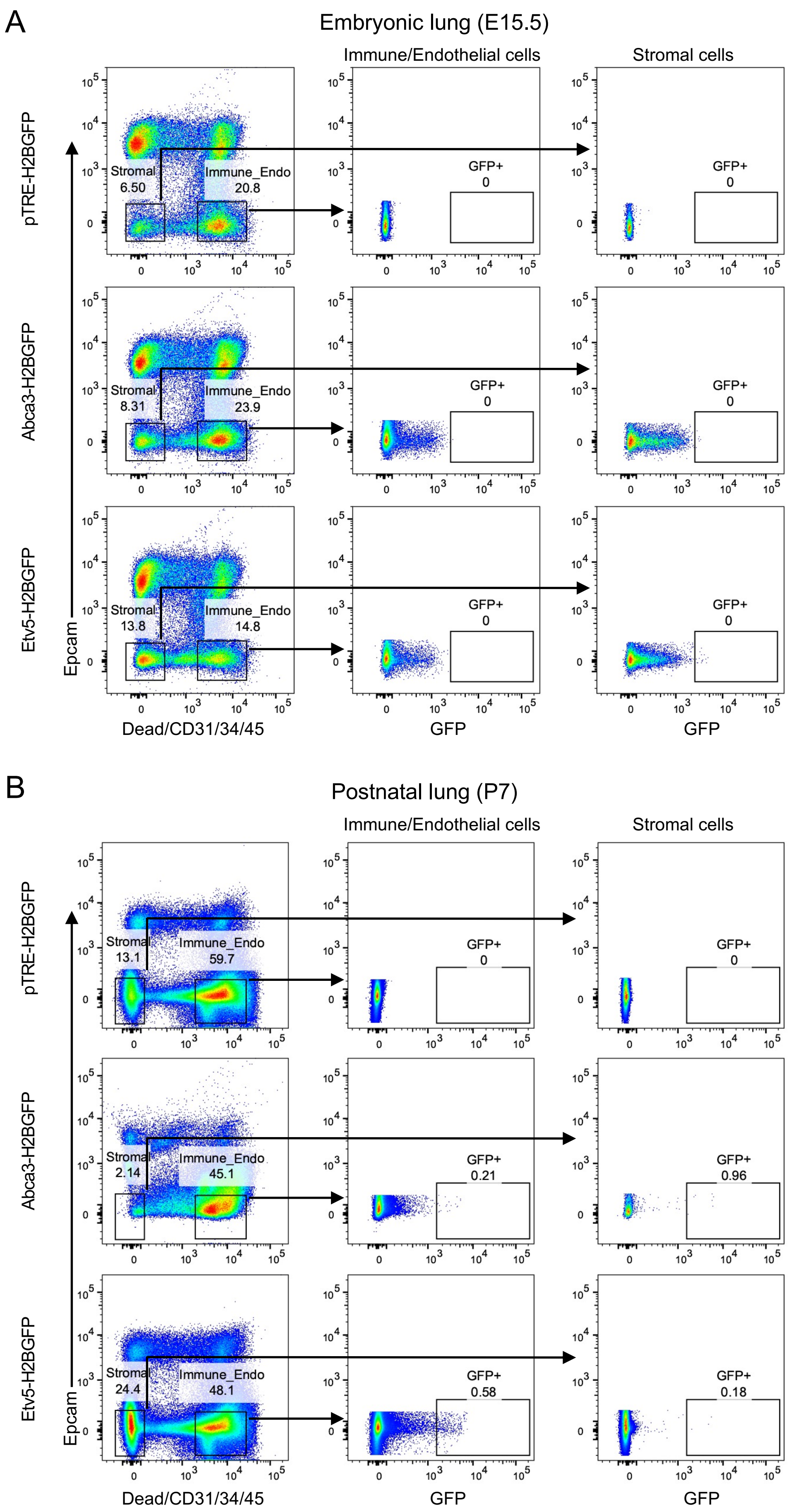

Figure S2. Representative flow cytometry plots showing the gating strategy for GFP^{+} cells in CD31/34/45^{+} mixed immune/endothelial cells and in double negative stromal cells in embryonic (A, E15.5) and postnatal (B, P7) *Abca3-H2BGFP* and *Etv5-H2BGFP* lungs, and littermate controls (*pTRE-H2BGFP*).

Figure S3

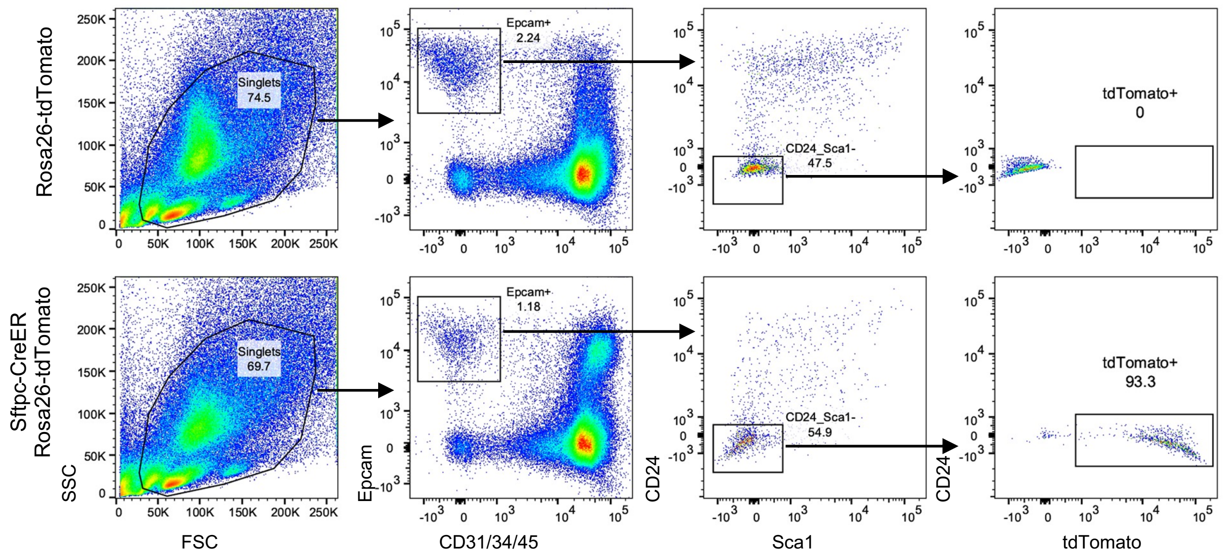

Figure S3. Representative flow cytometry plots showing the gating strategy for tdTomato+ cells in lungs from uninjured adult *Sftpc-CreER; Rosa26-tdTomato* and littermate control (*Rosa26-tdTomato*) mice after Tamoxifen induction.

Figure S4

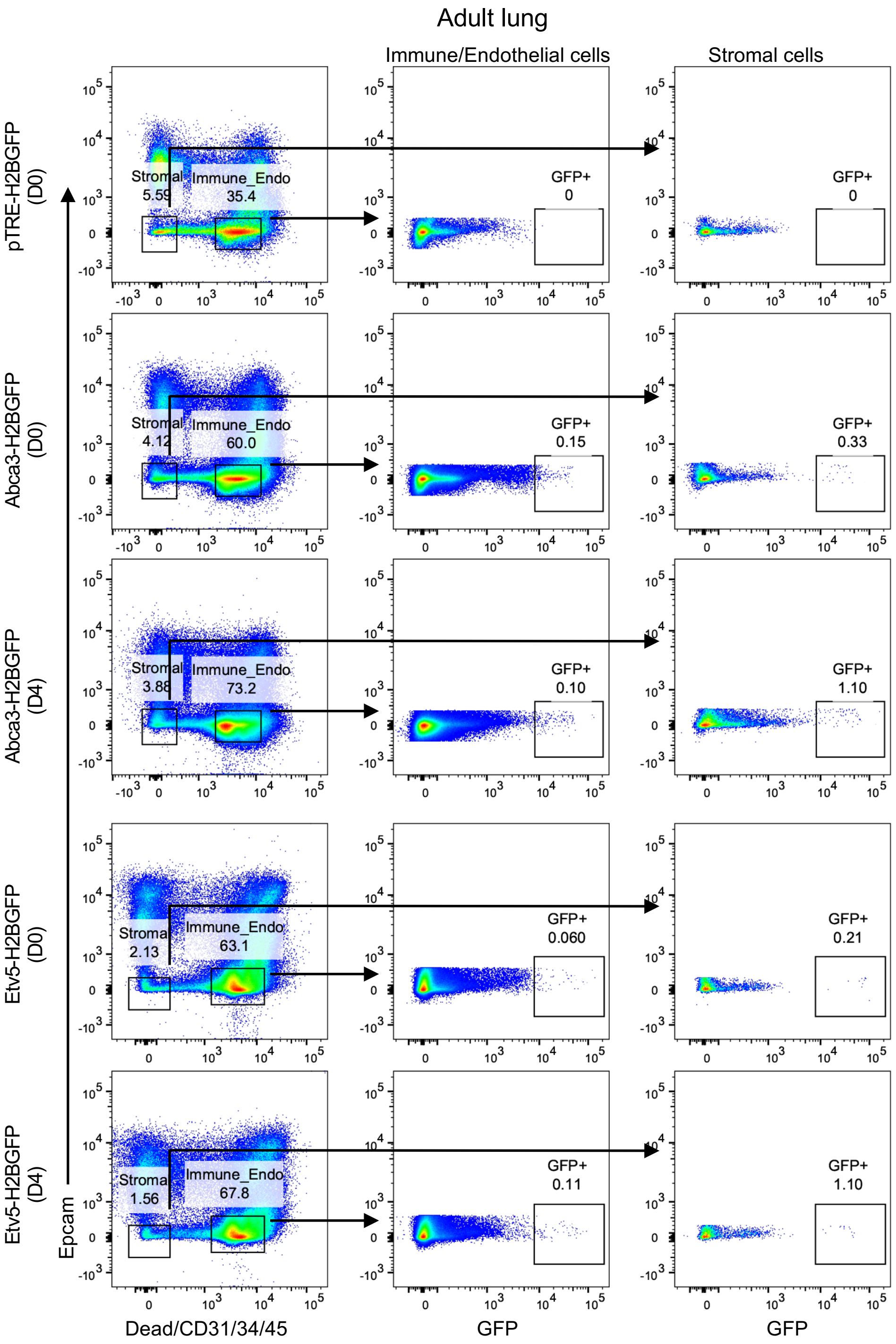

Figure S4. Representative flow cytometry plots showing the gating strategy for GFP^{+} cells in $CD31/34/45^{+}$ mixed immune/endothelial cells and in double negative stromal cells in lungs from adult *Abca3-H2BGFP* and *Etv5-H2BGFP*, and littermate control (*pTRE-H2BGFP*) mice before (D0) and 4 days after bleomycin injury (D4).
